# The Digital Diet Dilemma: Social Media’s Role in the Rise of Interest in Orthorexia

**DOI:** 10.1192/j.eurpsy.2025.1403

**Published:** 2025-08-26

**Authors:** J. Parkerson, C. Noureddine

**Affiliations:** 1 Psychiatry, Icahn School of Medicine at Mount Sinai, New York, United States

## Abstract

**Introduction:**

Since its inception in 1997, the term orthorexia nervosa (ON), derived from the Greek words for “right” and “appetite” (Donini et al, 2022), has been utilized to describe an obsession with “correct” or “healthy” eating. Despite numerous authors suggesting diagnostic criteria and the publication of several theoretical papers, consensus regarding a definition or even the existence of ON remains elusive (Dunn and Bratman, 2016). Conversely, anorexia nervosa (AN) has been recognized since the first iteration of the DSM (Bhattacharya et al, 2022; Dell’Osso et al 2016). Layperson awareness of these conditions has been increasing, with a notable increase in interest in “orthorexia” over the past several years, potentially influenced by popular culture and increased social media use (Sharma et al, 2023)

**Objectives:**

The purpose is to understand shifts in public interest as well as the ever-present influence of the online world on patients, especially those with disorder eating patterns.

**Methods:**

This study compared United States Google search trends for the terms “orthorexia” and “anorexia”. Using Google Trends data, we reviewed the average monthly search volume from January 2004 to March 2024. We analyzed the data to determine if there was a significant difference in search volume over time.

**Results:**

Google search data revealed a monthly search volume of ˜7000 queries for “orthorexia” in January 2004, and analysis of the volume revealed that there has been a significant and sustained increase in the searches for “orthorexia”. Additionally, searches for the term “anorexia” reached a height of 2.1 million in 2007, but have been steadily declining in the past several years.

While anorexia remains the more prominent search term, over time the queries for anorexia have decreased from their peak in the early 2000’s. The rise in the term orthorexia may be partly due to the proliferation of highly visual social media platforms, particularly YouTube and TikTok, where content creators often focus on healthy eating and lifestyle trends (Sharma et al, 2023).

Additionally, there is a possibility that orthorexia is being used on these platforms to describe a subset of AN. Some individuals with anorexia may present with behaviors resembling orthorexia, such as a fixation on healthy eating or a rigid adherence to specific dietary guidelines.

**Conclusions:**

The findings indicate a shift in the public’s interest in orthorexia and anorexia during the past several years, possibly influenced by increased social media use. Further research is needed to understand the implications of this trend on individuals’ attitudes towards healthy eating and body image.

**Disclosure of Interest:**

None Declared

